# Commentary: “Neuropsychological Assessment of Individuals with Brain Tumor: Comparison of Approaches Used in the Classification of Impairment”

**DOI:** 10.3389/fonc.2015.00188

**Published:** 2015-08-18

**Authors:** Kyle R. Noll, Joanna E. Fardell

**Affiliations:** ^1^Department of Neuro-Oncology, The University of Texas MD Anderson Cancer Center, Houston, TX, USA; ^2^Behavioural Sciences Unit proudly supported by Kids with Cancer Foundation, Kids Cancer Centre, Sydney Children’s Hospital and School of Women’s and Children’s Health, Faculty of Medicine, University of New South Wales, Sydney, NSW, Australia

**Keywords:** cognition, neuropsychology, assessment methods, brain tumors, neoplasms

Neurocognitive functioning (NCF) has been increasingly recognized as an important contributor to quality of life in patients with cerebral neoplasms. Accordingly, studies of NCF in this population have grown tremendously over the past few decades, and performance-based neuropsychological measures assessing diverse NCF domains are now routinely included in brain tumor clinical trials (1). However, little consensus exists regarding the prevalence of NCF impairment in this population, with Dwan et al. noting that rates reported in the literature vary from around 13% to over 90% (2). Numerous factors contribute to the widely differing impairment rates across studies, including tumor characteristics (histology, lesion size, and location) (3), patient demographics (socioeconomic status and age) (4), and treatments received (surgical resection and/or chemoradiation) (5).

The article by Dwan and colleagues highlights method variance as another important contributor to the variability in rates of NCF impairment reported. The authors adeptly point to a substantial lack of consistency in impairment classification schemas throughout the literature. Utilizing a few of the more commonly employed classification strategies in a sample of patients with brain tumors, they also demonstrate that classification of impairment based upon intra-individual comparison to estimated premorbid intellectual function differs from classification using published norms or matched controls. The authors then conclude that “this supports the need to interpret individuals’ neuropsychological test results in the context of their estimated premorbid IQ.” While we agree with Dwan et al. that consideration of premorbid functioning is important when interpreting neuropsychological test results, we believe that the approach utilized in their report may be prone to misclassification for reasons discussed below, and we briefly suggest an alternative that is potentially more statistically sound and robust.

In the analyses under discussion, the authors classify a test score as “impaired” if it falls at least one SD below the patient’s own premorbid intellectual function estimate, as determined by performance on a word-reading task. This method is economical and demands minimal statistical sophistication, making it attractive for use in clinical and research settings alike. However, as the authors briefly acknowledge, the approach risks elevating false positive (Type I) error rates for individuals at the upper tail of the premorbid intellectual distribution, as well as false negative errors (Type II) for those at the lower end of the distribution. Importantly, risk of error tends to increase with the number of tests included in a battery, which are numerous in routine neuropsychological practice, and when comparisons are made at the individual test level rather than domain level. A related issue involves differences between correlations among tests. Since tests of NCF vary in sensitivity, ceiling/floor levels, and domains of specificity, uniform correlations between measures of premorbid intellectual function and other tests are unlikely. This is problematic since it cannot be assumed that low-average estimated premorbid intellectual function entails that visual memory, processing speed, executive function, and performances within other domains should also be low average.

Fortunately, various methods exist to address aspects of these concerns. While a comprehensive delineation of the numerous techniques is beyond the scope of this paper, one popular method involves calculation of regression-based norms from healthy control data (6). With this approach, various variables can be used to build equations predicting expected performances for each test from estimates of premorbid intellectual function, age, education, and other factors known to influence NCF. With this method, the predicted (or expected) score can then be directly compared to the observed performance for an individual patient using inferential statistical analyses.

While this method has its own limitations and is by no means the only viable alternative, it has been shown to improve test sensitivity (6). Additionally, simulation studies show that variations of the technique have low classification error rates, even when control samples are relatively small (7). Some may object that these analyses are complicated and impractical. However, user friendly and freely available computer programs exist to facilitate the use of such methods (8, 9). These approaches can even be employed in studies lacking a healthy control group by substituting summary data gleaned from test manuals or previously published reports. Accordingly, consideration of including these and other novel analytical techniques into future studies regarding classification methodology is warranted, which may facilitate more precise prevalence estimates of NCF in patients with cerebral tumors.

## Conflict of Interest Statement

The authors declare that the research was conducted in the absence of any commercial or financial relationships that could be construed as a potential conflict of interest. The Associate Editor Kerrie Leanne McDonald and the Reviewer Eng-Siew Koh declare that, despite being affiliated to the same institution as author Joanna E. Fardell, the review process was handled objectively and no conflict of interest exists.
